# Future Perspectives: Mass Spectrometry for Spatial Localisation of Anti-Angiogenic Oil Palm Compounds

**DOI:** 10.3390/ijms27083351

**Published:** 2026-04-08

**Authors:** Fatimah Zachariah Ali, Norfazlina Mohd Nawi, Wijenthiran Kunasekaran, Tan Li Jin, Lee Siew Ee, Nazia Abdul Majid

**Affiliations:** 1 Division of Surgery & Interventional Science, University College London, Charles Bell House, 43–45 Foley St, London W1W 7TS, UK; fatimah.ali.16@ucl.ac.uk; 2 Institute of Biological Sciences, Faculty of Science, Universiti Malaya, Kuala Lumpur 50603, Malaysia; 3 Nano Biologics Research Centre (NBRC), WARI Technologies Sdn Bhd, 2A-2, Galleria Cyberjaya, Jalan Teknokrat 6, Cyber 5, Cyberjaya 63000, Malaysia; wijen@wari.tech (W.K.); lijin@wari.tech (T.L.J.); siewee@wari.tech (L.S.E.)

**Keywords:** angiogenesis, colorectal cancer, oil palm bioactive compounds, palm oil mill effluent (POME), mass spectrometry imaging (MSI), tumour microenvironment, precision nutraceutical oncology

## Abstract

Angiogenesis is a spatially regulated hallmark of colorectal cancer (CRC) progression, yet current analytical frameworks fail to resolve how nutraceutical bioactive compounds interact with angiogenic signalling within the heterogeneous tumour microenvironment. This review advances a central hypothesis: that the spatial localisation of palm oil mill effluent (POME)-derived bioactive compounds within CRC tumour tissues is predictive of their functional anti-angiogenic activity. POME—the largest waste stream of palm oil processing—contains a chemically diverse array of bioactives, including tocotrienols, phenolics, carotenoids, and fatty acids, with reported antioxidant, anti-inflammatory, and anti-angiogenic properties. However, the existing evidence is predominantly derived from bulk in vitro analyses, limiting mechanistic conclusions about compound behaviour within spatially organised tumour architectures. To address this gap, we propose an integrated framework positioning mass spectrometry imaging (MSI)—across matrix-assisted laser desorption/ionisation (MALDI), desorption electrospray ionisation (DESI), and secondary ion mass spectrometry (SIMS) platforms—as the analytical bridge between compound localisation and angiogenic function. By enabling the label-free, spatially resolved co-localisation of POME-derived compounds with key angiogenic mediators, including VEGF, HIF-1α, and NF-κB, within intact CRC tissues, MSI provides a mechanistic platform that transcends the limitations of conventional molecular analyses. A four-component translational roadmap is outlined, encompassing POME bioactive profiling, spatial compound mapping, angiogenic co-localisation analysis, and functional validation. Critically, the existing evidence on oil palm-derived bioactives is appraised with respect to study quality, mechanistic depth, and translational limitations, identifying the most analytically tractable candidate compounds for spatial investigation. Collectively, this framework positions POME valorisation within a precision nutraceutical oncology paradigm, offering a spatially informed strategy for anti-angiogenic intervention in CRC while simultaneously addressing the environmental burden of palm oil processing waste.

## 1. Introduction

Colorectal cancer (CRC) remains one of the most prevalent and lethal malignancies worldwide [1], with tumour-associated angiogenesis representing a critical driver of disease progression, metastatic dissemination, and therapeutic resistance [2,3]. The formation of an aberrant and spatially heterogeneous vascular network within the CRC tumour microenvironment (TME) not only sustains tumour growth under hypoxic conditions but also facilitates immune evasion and limits the efficacy of systemic therapies [4]. Although the molecular regulators of angiogenesis, principally vascular endothelial growth factor (VEGF), hypoxia-inducible factor-1α (HIF-1α), and nuclear factor-κB (NF-κB), have been extensively characterised [5,6], current anti-angiogenic pharmacological strategies are constrained by dose-limiting toxicity, acquired resistance, and an inability to account for the spatial complexity of signalling within heterogeneous tumour architectures. These limitations underscore a fundamental unmet need: analytical and therapeutic frameworks capable of resolving angiogenic signalling not merely at the molecular level but within its native spatial and microenvironmental context.

Nutraceutical bioactive compounds have gained increasing attention as modulators of cancer-associated signalling pathways, offering a potentially safer and more physiologically integrated alternative or complement to pharmacological intervention [7]. Among candidate bioresources, oil palm (*Elaeis guineensis*) processing by-products, particularly palm oil mill effluent (POME), represent an abundant yet critically underexplored source of chemically diverse bioactives, including tocotrienols, phenolic compounds, carotenoids, and fatty acids. POME is the largest volumetric waste stream generated in palm oil processing and is currently managed primarily through environmentally burdensome treatment systems that discard its bioactive constituents without recovery [8]. Critically, while isolated oil palm-derived compounds have been individually investigated for antioxidant, anti-inflammatory, and anti-angiogenic properties, no systematic framework exists for evaluating POME as a compositionally complex bioresource within the spatially organised context of the CRC tumour microenvironment. This represents a substantive gap that existing reviews have not addressed.

A central limitation of current nutraceutical oncology research is its reliance on bulk biochemical and molecular analyses, including ELISA, PCR, and standard immunohistochemistry, which, while informative, cannot resolve how bioactive compounds distribute within heterogeneous tissue architectures or whether their presence correlates spatially with sites of active angiogenic signalling. This is particularly consequential given that angiogenesis in CRC is not a spatially uniform process. Hypoxic niches, vascular remodelling zones, and stromal–tumour interfaces each represent distinct microenvironmental contexts in which angiogenic mediators operate differentially. Without spatial resolution, it is impossible to determine whether a nutraceutical compound reaches, accumulates within, or functionally engages these angiogenic niches, a fundamental requirement for establishing the mechanism of action.

We therefore propose a conceptual framework centred on the hypothesis that the spatial localisation of POME-derived bioactive compounds within CRC tumour tissues is predictive of their functional anti-angiogenic activity. This hypothesis reframes the evaluation of nutraceutical compounds from descriptive bioactivity profiling toward spatially informed mechanism-of-action investigation. To operationalise this framework, we position mass spectrometry imaging (MSI), encompassing matrix-assisted laser desorption/ionisation (MALDI), desorption electrospray ionisation (DESI), and secondary ion mass spectrometry (SIMS) platforms, as the analytical cornerstone, given its unique capacity for the label-free, high-resolution spatial mapping of metabolites, lipids, and bioactive compounds directly within intact tissue sections. Critically, MSI enables simultaneous co-localisation of exogenous compounds with endogenous angiogenic markers, allowing spatial correlation analyses that are inaccessible to conventional approaches.

**Figure 1:**
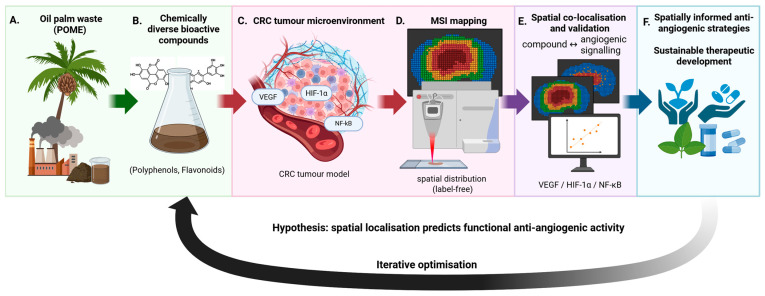
**Spatially resolved framework for investigating anti-angiogenic oil palm-derived compounds in colorectal cancer.** Oil palm waste, particularly palm oil mill effluent (POME), represents a sustainable source of chemically diverse bioactive compounds (**A**,**B**). These compounds are introduced into colorectal cancer (CRC) models characterised by spatially heterogeneous tumour microenvironments, including hypoxic regions and angiogenic niches regulated by pathways such as VEGF, HIF-1α, and NF-κB (**C**). Mass spectrometry imaging (MSI) enables label-free, spatial mapping of compound distribution alongside endogenous angiogenic markers within intact tissue architecture (**D**). Integration of these datasets allows spatial co-localisation and correlation analyses, which can be further validated, linking compound localisation to the modulation of angiogenic signalling (**E**). This framework supports the development of spatially informed, sustainable anti-angiogenic strategies and enables iterative optimisation of candidate compounds for CRC therapy (**F**).

The forward-looking roadmap proposed in this review comprises four integrated components (Figure 1): (i) systematic chemical profiling of POME-derived bioactive diversity; (ii) spatially resolved compound mapping within CRC tissue models using MSI; (iii) co-localisation analysis, with key angiogenic mediators including VEGF, HIF-1α, and NF-κB; and (iv) functional validation of spatially associated anti-angiogenic effects. Each component is designed to be sequentially testable, collectively constituting a translational pipeline from a waste-derived bioresource to a spatially validated anti-angiogenic candidate. This framework does not merely describe what is known about POME or MSI in isolation—it advances a testable proposition about how spatial compound distribution governs nutraceutical efficacy, with direct implications for the development of precision nutraceutical oncology strategies in CRC.

## 2. Overview of Oil Palm Bioactive Components

**Figure 2:**
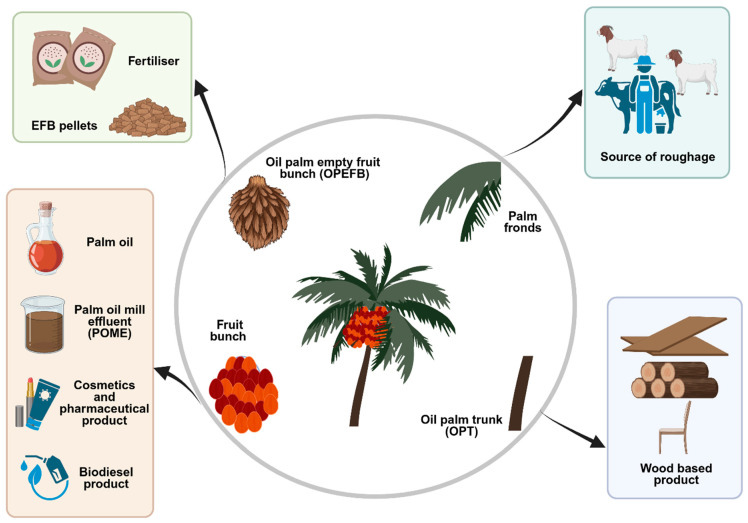
**Value-added applications of oil palm biomass and by-products.** Schematic representation of the oil palm (Elaeis guineensis) biomass utilisation cycle and its diverse value-added products, derived from fresh fruit bunches and associated processing residues. Oil palm processing generates multiple biomass streams, including empty fruit bunches (EFBs), EFB pellets, palm fronds, oil palm trunks (OPTs), crude palm oil, and palm oil mill effluent (POME). These materials can be further converted into a wide range of industrial and biotechnological products, such as fertilisers, biodiesel, cosmetic and pharmaceutical ingredients, livestock feed or roughage, and wood-based materials (created with BioRender).

Oil palm is a major agricultural crop in Indonesia and Malaysia and plays a crucial role in the countries’ economic development [9,10]. During palm oil processing, approximately 21% of fresh fruit bunches (FFBs) are converted into crude palm oil, while the remaining 79% is generated as by-products and residues. These include palm kernel cake (PKC), palm-pressed fibre (PPF), oil palm empty fruit bunches (OPEFB), palm oil mill effluent (POME), palm kernel shells, oil palm trunks, and oil palm leaves, which collectively raise significant sustainability and environmental concerns [11]. In response to these challenges, many studies have explored the valorisation of oil palm by-products into value-added products, such as organic fertilisers [12], EFB pellets for power generation [13], sources of roughage for animal feed, and wood-based materials [14] (Figure 2). Despite these efforts, certain by-products, particularly OPEFB and POME, remain underutilised or are still disposed of in environmentally unsustainable ways.

Among these by-products, OPEFB contributes to greenhouse gas emissions and potential groundwater contamination, while POME, when discharged at high temperatures, causes thermal shock and environmental pollution [15]. Scalable and effective alternatives for managing these waste streams remain limited, and these challenges are expected to intensify alongside continued expansion of the palm oil industry.

As the most voluminous waste stream generated during palm oil processing [16], POME originates from sterilisation, extraction, and clarification stages and presents considerable operational, economic, and environmental burdens for wastewater management systems [17,18]. With global palm oil demand projected to rise, POME generation is expected to increase correspondingly, underscoring the urgent need for sustainable treatment strategies centred on resource recovery and bioactive compound extraction [19]. Critically, however, existing approaches to POME management have overwhelmingly focused on remediation and pollutant reduction, with comparatively little systematic attention directed toward the recovery and characterisation of its bioactive constituents. This represents a substantive and under-addressed gap in the literature.

**Figure 3:**
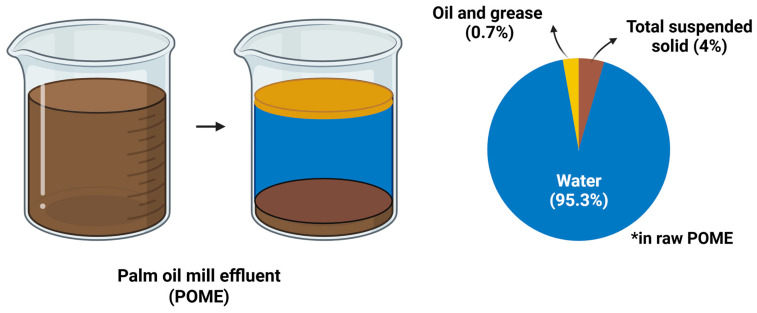
**Compositional distribution of major constituents in raw palm oil mill effluent (POME).** Percentage composition of raw palm oil mill effluent (POME) generated during palm oil processing. Raw POME is predominantly composed of water (~95.3%), with smaller fractions of total suspended solids (~4%) and oil and grease (~0.7%). The high water content contributes to the liquid and viscous nature of POME, while suspended solids and residual oil originate from organic plant materials and lipid residues released during the sterilisation, extraction, and clarification stages of palm oil milling (created with BioRender). * Data are derived from raw POME.

Figure 3 illustrates the compositional profile of raw POME, which is predominantly composed of water (94.2–95.3%), followed by total suspended solids (4.0–5.0%) and oil/grease (0.7–0.8%) [20]. This complex composition contributes to the characteristic thick, brownish appearance of POME, which exists as a viscous and colloidal suspension [21]. The dark brown coloration of POME is primarily attributed to the presence of tannins, lignin, and phenolic compounds [22], as well as carotene and pectin [23]. Beyond their physicochemical significance, these constituents are biologically active molecules with potential nutraceutical and pharmacological relevance. In particular, phenolic compounds and carotenoids are widely recognised for their antioxidant [24,25], anti-inflammatory, and anti-cancer properties [26,27], suggesting that POME may represent an underexplored reservoir of recoverable bioactives.

Quantitative analyses further support this potential: extractable oil has been recovered from POME at concentrations of approximately 1710–3280 mg/L, carotene content has been reported at approximately 400 ppm, vitamin E homologues at approximately 706–743 ppm [28], and total phenolic content at approximately 1163–3545 μg/g [29]. Advanced extraction strategies have demonstrated a recovery of up to 85% of polyphenols using a synergistic green extraction system comprising 0.25 M 1-octanol and 0.05 M Aliquat 336 [30]. These findings confirm that POME contains measurable and recoverable levels of bioactive compounds. However, several important caveats must be acknowledged. First, reported bioactive concentrations vary considerably across studies, likely reflecting differences in mill type, feedstock quality, processing conditions, and seasonal variation, sources of variability that are frequently underreported and limit cross-study comparability. Second, these compounds are not present in POME at concentrations equivalent to their primary botanical sources; the translational significance of POME lies in its potential as a scalable, recoverable bioresource rather than as a concentrated extract. Third, the extent to which conventional POME treatment processes, including anaerobic ponding and digestion, degrade or structurally alter these bioactives prior to any recovery attempt has not been systematically evaluated, representing a critical gap in the valorisation pipeline.

Table 1 summarises the major bioactive constituents identified in raw POME, including tannins, lignin, phenolics, carotenoids, pectin, vitamin E, and fatty acids, alongside their reported biological mechanisms and prospective translational applications. While this compositional profile is promising, it is important to note that the biological activity data associated with several of these constituents has largely been established using purified compounds from non-POME sources, including other plant species, bacteria, and fungi. Direct evidence of equivalent bioactivity for POME-derived fractions remains limited and should not be assumed without POME-specific extraction, characterisation, and bioactivity validation. This distinction is critical for accurately representing the current state of evidence and identifying where further investigation is most urgently needed.

Conventional POME treatment strategies primarily target the degradation or removal of organic matter and suspended solids to meet discharge standards, inadvertently discarding compounds of potential therapeutic value. Re-evaluating these constituents through a nutraceutical and pharmaceutical lens aligns with circular bioeconomy principles and offers a compelling opportunity to transform an environmental burden into a high-value medicinal resource.

Importantly, this reframing distinguishes the present review from prior work: whereas existing reviews have focused predominantly on isolated oil palm-derived compounds such as tocotrienols and palm phenolics, the present work addresses POME as a compositionally complex and undercharacterised bioresource and situates its bioactive potential within a spatially resolved tumour microenvironment framework. Specifically, we propose that MSI provides the analytical basis for determining whether POME-derived compounds, once administered in preclinical CRC models, localise to angiogenic niches and correlate spatially with key signalling mediators, thereby linking compound distribution to functional anti-angiogenic activity in a manner that bulk analyses cannot achieve. 

## 3. Integration with Tumour Angiogenesis Models

Angiogenesis is a fundamental hallmark of cancer and plays a critical role in tumour progression. The formation of new blood vessels from pre-existing vasculature enables tumours to secure an adequate supply of oxygen and nutrients [54], thereby supporting sustained growth beyond a limited size threshold. Beyond nutrient delivery, neovascularisation also provides a structural and functional conduit for tumour cell intravasation and dissemination, facilitating metastasis to distant tissues and organs. The activation of the “angiogenic switch” is therefore a pivotal event in tumour development, regulated by a complex interplay of pro-angiogenic factors, including angiopoietins, VEGF, transforming growth factor-β1 (TGFβ1)*,* HIF-1α, matrix metalloproteinases (MMPs), and Platelet-Derived Growth Factor (PDGF) [55]. Critically, this signalling network is not spatially uniform; angiogenic activity is concentrated within discrete microenvironmental niches, including hypoxic regions, invasive fronts, and stromal interfaces, a spatial organisation that has direct implications for how nutraceutical compounds must be evaluated if their anti-angiogenic potential is to be meaningfully assessed.

Understanding how POME-derived compounds influence angiogenic signalling is therefore central to assessing their therapeutic potential and requires experimental models capable of recapitulating the spatial and functional complexity of the tumour vasculature. In vitro approaches include endothelial cell proliferation, migration, and tube formation assays [56], as well as three-dimensional co-culture spheroid systems that replicate tumour–endothelial interactions [57]. These models are primarily used to examine the modulation of key angiogenic mediators such as VEGF, TGFβ and angiopoietins as well as related downstream signalling pathways. However, it should be noted that in vitro systems lack the spatial tissue architecture necessary to determine how bioactive compounds distribute relative to angiogenic structures, which is a limitation that motivates the MSI-based framework proposed in this review.

For in vivo validation, commonly applied systems include the chicken chorioallantois membrane (CAM) assay [58], the dorsal air sac model [59], and transgenic mouse tumour models such as the VEGFR2-Luc Murine Breast Cancer Model [60] as well as syngeneic mouse models like the 4T1 model [61]. These platforms allow assessment of micro vessel formation and tumour-associated vascular development within an intact biological environment and, importantly, generate tissue specimens amenable to downstream spatial molecular analysis, including MSI.

**Figure 4:**
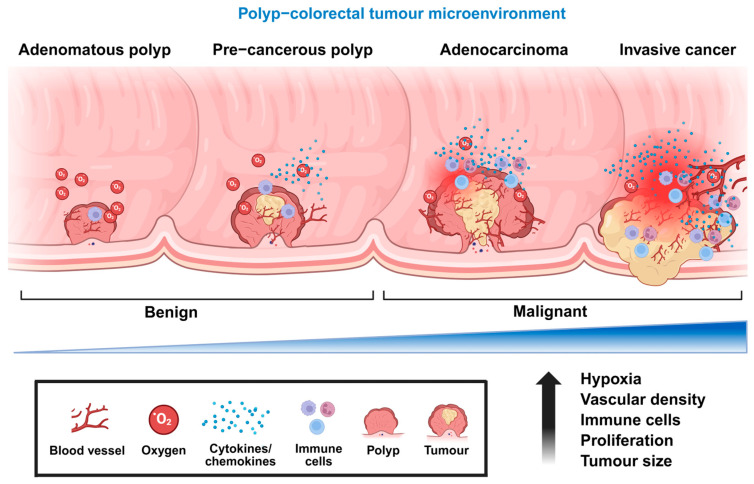
**Multistep progression from colorectal polyp to invasive carcinoma within the tumour microenvironment.** Schematic illustration of the multistep progression of colorectal tumorigenesis, beginning with benign adenomatous polyps and advancing through precancerous lesions to malignant adenocarcinoma and invasive colorectal cancer. During this progression, the tumour microenvironment undergoes dynamic changes characterised by increasing hypoxia, vascular density, immune cell infiltration, and cellular proliferation. These alterations collectively promote tumour growth and facilitate the transition from benign to malignant states. The diagram also highlights the involvement of blood vessels, oxygen gradients, immune cells, and cytokine/chemokine signalling within the evolving tumour microenvironment.

This review places particular emphasis on angiogenesis in colorectal cancer, supported by the increasing incidence of CRC among adults younger than 50 years, which has become an emerging public health concern [62]. The rising trend in early-onset CRC highlights the urgent need for preventive and therapeutic strategies targeting tumour progression at its earliest stages. Early detection remains essential for improving clinical outcomes, with molecular markers associated with colorectal polyps, including adenomas and serrated polyps, offering potential targets for early intervention [63]. Most colorectal cancers arise through a multistep polyp–carcinoma sequence [64], and polyp enlargement has been correlated with increased vascularisation, indicating active microvascular support even at premalignant stages [65]. As angiogenesis is recognised as a hallmark of tumour growth [66], enhanced vascular development may contribute to polyp expansion and progression toward malignant transformation (Figure 4).

Targeting angiogenic pathways therefore represents a promising strategy not only for established CRC but also for early premalignant lesions, where intervention may prevent malignant transformation. In this context, POME-derived bioactive compounds, with reported modulatory effects on VEGF, HIF-1α, and NF-κB signalling, represent candidates for evaluation across this disease continuum. However, whether these compounds reach and engage angiogenic niches within premalignant or tumour tissues remains unknown, precisely because current experimental approaches do not resolve compound distribution within tissue architecture.

In colorectal cancer research, preclinical models that mimic polyp formation and advanced tumour development are commonly induced in mice using azoxymethane (AOM) and dextran sulphate sodium (DSS), either individually [67] or in combination [68]. Additional models employ 1,2-dimethylhydrazine (DMH) [69] or naltrexone (NTX) treatment [70], which exhibit tumour-associated angiogenesis and provide suitable platforms for evaluating anti-angiogenic interventions. Among these, the AOM/DSS model is particularly well-suited to the framework proposed in this review, as it generates tumours with spatially heterogeneous microenvironments, including hypoxic niches and active angiogenic zones within tissue specimens, which are directly compatible with FFPE processing and downstream MSI analysis. Strategic integration of these chemical induction protocols may further refine preclinical models to better represent the continuum from polyp formation to invasive carcinoma, providing robust and spatially tractable platforms for assessing the anti-angiogenic effects of POME-derived compounds in conjunction with spatial molecular profiling.

## 4. Molecular Basis of Angiogenesis in Colorectal Cancer Development

**Figure 5:**
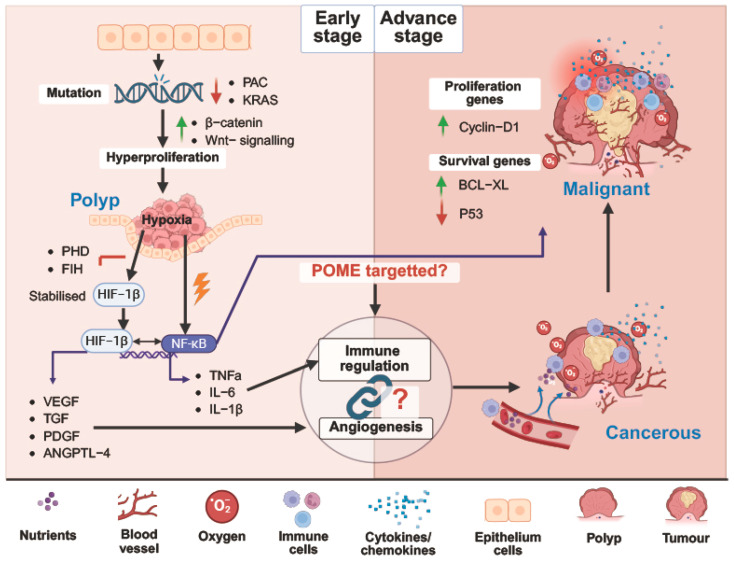
**Molecular signalling pathways involved in colorectal cancer progression and potential targets of POME−derived bioactive compounds.** Schematic overview of molecular events driving colorectal cancer progression from adenomatous polyp to malignant tumour. Early genetic mutations, including APC and KRAS alterations, activate β−catenin/Wnt signalling, leading to epithelial hyperproliferation and polyp formation. Hypoxia within the polyp stabilises HIF−1, which, together with NF−κB activation, promotes inflammatory cytokine production (TNF−α, IL−6, IL−1β) and induces pro-angiogenic factors such as VEGF, TGF, PDGF, and ANGPTL−4. Subsequent dysregulation of proliferation and survival pathways, including increased cyclin D1 and BCL−XL and reduced p53 activity, supports tumour growth and malignant transformation. The diagram also highlights potential intervention points where POME−derived bioactive compounds may modulate immune regulation and angiogenic signalling. Arrows indicate an increase or decrease in molecular expression. “?” denotes the potential or unclear link between immune regulation and angiogenesis.

CRC develops through a stepwise process from adenoma to invasive carcinoma, driven by sequential genetic mutations, including loss of the adenomatous polyposis coli (APC) tumour suppressor gene, activation of the KRAS oncogene, and loss of the TP53 [71]. The APC gene, located on chromosome 5q, negatively regulates the β-catenin/Wnt signalling pathway, which governs cell growth and differentiation. Mutations in APC disrupt the β-catenin destruction complex, comprising APC, Axin/Axin2, GSK-3β, and CK1, allowing β-catenin to accumulate, translocate to the nucleus, and activate the transcription of proliferative target genes. Subsequent activating mutations in KRAS, particularly at codons 12 and 13, promote colorectal polyp formation [72], while loss of p53 [73], upregulation of BCL-XL [74] and increased cyclin D1 activity [75] drive malignant transformation and progression to invasive carcinoma (Figure 5).

In parallel with these genetic alterations, angiogenesis becomes progressively activated during colorectal tumorigenesis. The transition from normal colonic epithelium to adenoma and ultimately carcinoma is accompanied by disruption of the physiological balance between pro- and anti-angiogenic signals, resulting in sustained neovascularisation within the polyp-tumour microenvironment [2].

Among the key mediators, VEGF plays a central role. Upon binding to VEGFR1 and VEGFR2, VEGF activates multiple downstream cascades: the PI3K–Akt pathway enhances endothelial cell survival and vascular permeability; the PI3K–mTORC2 and Raf–MEK–ERK pathways promote proliferation; and the Src–FAK pathway regulates cytoskeletal reorganisation and motility [76]. Overexpression of VEGF and VEGFR2 is frequently observed in colorectal tumours and is strongly associated with increased micro vessel density, tumour aggressiveness, and poor clinical prognosis.

Hypoxia is another major driver of angiogenesis in CRC. Rapid tumour cell growth outstrips the surrounding vasculature, creating oxygen-deficient microenvironments. Notably, adenomas have been reported to be more hypoxic than hyperplastic polyps and serrated lesions [77]. Under hypoxic conditions, the suppression of prolyl hydroxylase domain proteins (PHDs) and factor inhibiting HIF (FIH) prevents HIF-1α degradation, enabling its stabilisation, nuclear translocation, and activation of hypoxia-responsive transcriptional programmes [78]. Beyond oxygen-dependent regulation, HIF-1α activity is further modulated by growth factors via PI3K/Akt/mTOR and Ras/Raf/MEK/ERK axes, by cytokines via JAK/STAT signalling, and by Wnt/β-catenin, Notch, and NF-κB pathways [79]. Together, these inputs regulate the transcription of genes driving angiogenesis, inflammation, metabolic reprogramming, and therapeutic resistance.

The hypoxia–HIF-1α–VEGF axis represents a fundamental regulatory mechanism in tumour vascular development. Under hypoxic conditions, HIF-1α binds the regulatory region of the VEGF gene, inducing its transcription and promoting endothelial cell migration toward hypoxic areas to form new blood vessels [80]. Hypoxia additionally upregulates other angiogenic genes, including Smad7, Jun, IL-8, CXCR-4, PDGF-A, TGF-A, and ANGPTL-4 in HCT-116 cells [81], further amplifying the pro-angiogenic programme.

An immunological microenvironment is also present within colorectal polyps, although the mechanistic roles of many immune cell subsets remain less well defined than in established CRC. Regulatory T cells and dendritic cells have been associated with polyp lesions, with Treg infiltration increasing during malignant transformation and conventional adenomas exhibiting a reduction in mature dendritic cells [82]. Assessment of inflammatory cell phenotypes in colonic adenomas has demonstrated the presence of T and B lymphocytes, NK cells, macrophages, mast cells, neutrophils, and plasma cells within adenomatous tissue [83]. However, whether immune modulation within polyps actively drives angiogenic pathway activation, or conversely, whether angiogenic signalling reshapes immune regulation, remains to be clarified.

In established CRC, inflammatory signalling promotes angiogenesis via the NF-κB pathway, a key molecular link between inflammation and neovascularisation. Upon activation, NF-κB translocates to the nucleus and induces the transcription of genes involved in angiogenesis and tumour growth [84], including pro-inflammatory cytokines (IL-1β, IL-6, MCP-1), adhesion molecules (ICAM-1), and pro-angiogenic mediators (VEGF, MMP-9, IL-8) [85]. Under hypoxic conditions, LDL has further been shown to enhance interactions among TNF-α, NF-κB, HIF, and VEGF signalling in endothelial cells, reinforcing angiogenic responses [86]. Given that colorectal polyps may also develop hypoxic regions, similar immune–angiogenic crosstalk plausibly occurs during early premalignant stages (Figure 5), though direct evidence in this context remains limited.

Collectively, CRC-associated angiogenesis is governed by interconnected hypoxia, inflammatory, and growth factor–dependent signalling networks. These pathways are not spatially uniform; rather, their activity is concentrated within discrete microenvironmental niches, hypoxic zones, invasive fronts, and stromal interfaces, whose spatial organisation cannot be resolved by conventional bulk molecular analyses. This spatial heterogeneity is directly relevant to the evaluation of POME-derived bioactive compounds, as modulation of VEGF, HIF-1α, or NF-κB signalling by nutraceutical compounds can only be meaningfully interpreted if the location of both the compound and its putative molecular targets within the tumour microenvironment is known. MSI provides the analytical means to resolve this spatial context, enabling direct investigation of whether POME-derived compounds co-localise with angiogenic signalling niches and whether their distribution correlates with functional pathway modulation, the central proposition of this review.

## 5. Anti-Angiogenic and Anti-Cancer Effects of Oil Palm Components

**Figure 6:**
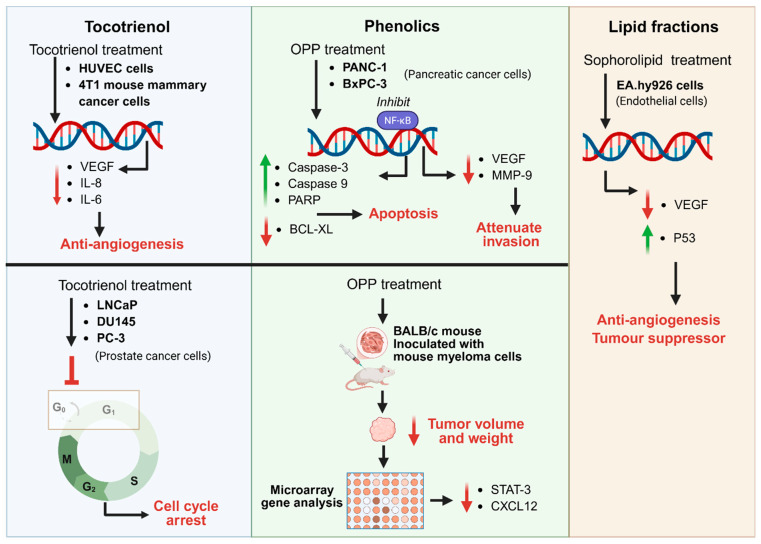
**Anti−angiogenic and anti−tumour mechanisms of oil palm−derived bioactive compounds.** Overview of molecular pathways targeted by major oil palm-derived bioactive compounds, including tocotrienols, phenolic compounds, and lipid fractions. Tocotrienols suppress angiogenesis by reducing pro-inflammatory and pro-angiogenic mediators such as VEGF, IL−6, and IL−8, and induce cell cycle arrest in cancer cells. Oil palm phenolics inhibit NF−κB signalling, promote apoptosis through activation of caspase pathways and PARP cleavage, and attenuate tumour invasion by reducing VEGF and MMP−9 expression. In vivo studies also show that OPP treatment reduces tumour volume and weight through downregulation of STAT3 and CXCL12 gene expression. Lipid fractions, including sophorolipids, contribute to anti-angiogenic effects by decreasing VEGF levels and enhancing tumour suppressor signalling such as p53. Collectively, these mechanisms demonstrate that oil palm-derived bioactive compounds modulate inflammatory, apoptotic, and angiogenic pathways to inhibit tumour growth and vascular development. Green upward arrows indicate an increase, red downward arrows indicate a decrease, and blunt-ended lines indicate inhibition.

Given the pivotal role of angiogenesis in colorectal tumour initiation and progression, bioactive constituents derived from oil palm have attracted considerable interest for their potential anti-angiogenic and anti-cancer properties. Tocotrienol, phenolic, and lipid fractions have been reported to exhibit potential anti-cancer, anti-inflammatory, and anti-angiogenic activities, with key molecular targets including VEGF, HIF-1α, NF-κB, and related inflammation-associated mediators (Figure 6). However, a critical appraisal of this evidence is warranted before these findings can be meaningfully interpreted in the context of CRC therapeutic development.

### 5.1. Tocotrienols

Among oil palm-derived bioactives, tocotrienols, particularly the tocotrienol-rich fraction (TRF), represent the most extensively studied class. Tocotrienols have been reported to suppress angiogenesis by reducing pro-inflammatory and pro-angiogenic mediators, including VEGF, IL-6, and IL-8, and to induce cell cycle arrest in cancer cell lines [87,88]. However, most mechanistic studies were conducted between 2006 and 2019 using in vitro cell line models, predominantly HeLa, HCT116, and HT-29, under standard culture conditions that do not recapitulate the hypoxic, spatially heterogeneous tumour microenvironment relevant to CRC in vivo. Critically, reported anti-angiogenic effects are largely inferred from changes in downstream molecular markers, such as VEGF protein expression or NF-κB activation, rather than confirmed through functional angiogenesis assays such as tube formation inhibition, ex vivo aortic ring models, or in vivo vascular density quantification. Without such functional validation, the modulation of molecular markers alone cannot be interpreted as evidence of meaningful anti-angiogenic activity. Furthermore, several studies report inhibitory effects only at supraphysiological compound concentrations unlikely to be achieved in vivo following oral administration, a pharmacokinetic limitation that is rarely addressed in the source literature but has direct implications for therapeutic feasibility.

### 5.2. Oil Palm Phenolics

Oil palm phenolics (OPPs) have been reported to inhibit NF-κB signalling, promote apoptosis via caspase activation and PARP cleavage, and attenuate tumour invasion through downregulation of VEGF and MMP-9 [89,90]. In vivo studies further indicate that OPP treatment reduces tumour volume through the downregulation of STAT3 and CXCL12 gene expression. While these findings are more translationally relevant than in vitro data alone, the number of independent in vivo replications remains limited, and the spatial distribution of phenolic compounds within tumour tissues—and whether they reach angiogenic niches at therapeutically relevant concentrations—has not been investigated. This represents a fundamental mechanistic gap that bulk tissue analyses cannot resolve.

### 5.3. Lipid Fractions

Lipid fractions, including sophorolipids, have been reported to contribute to anti-angiogenic effects by decreasing VEGF levels and enhancing tumour suppressor signalling, including p53 activation [91]. However, the evidence base for lipid-mediated anti-angiogenic activity in oil palm-specific contexts is comparatively sparse, and the isoform-dependent pro- or anti-angiogenic duality of fatty acids, documented in non-palm sources, has not been systematically characterised for POME-derived lipid fractions. This ambiguity warrants caution in extrapolating anti-angiogenic conclusions without isoform-specific characterisation.

### 5.4. Carotenoids

Carotenes have been shown to prevent oxidative degradation during palm oil processing through free radical scavenging [92], and their antioxidant properties may confer cellular protection against ROS-mediated carcinogenesis. However, direct evidence for the anti-angiogenic activity of oil palm-derived carotenoids in tumour models is limited. The inference from antioxidant capacity to anti-angiogenic function requires mechanistic substantiation, as ROS also serve context-dependent regulatory roles in angiogenic signalling. The anti-angiogenic potential of oil palm carotenoids therefore remains a hypothesis rather than an established finding.

Across all compound classes, the findings are inconsistent. While some studies demonstrate clear suppression of VEGF and HIF-1α, others report indirect or context-dependent effects that vary with the experimental model, compound concentration, and endpoints evaluated. This variability complicates direct comparison and is rarely discussed critically in the source literature. The predominance of single-laboratory studies has limited independent replication, and reliance on a narrow range of cell line models collectively restricts the generalisability of the current conclusions. More broadly, no study to date has investigated how any of these oil palm-derived compounds distribute within intact CRC tumour tissues, whether they reach biologically relevant regions such as angiogenic hotspots or hypoxic niches, or how their intratissue localisation relates to observed molecular effects. This spatial dimension is entirely absent from the current evidence base. 

High-dose TRF administration produced no significant hepatic or renal toxicity in murine models, as assessed by ALT, ALP, creatinine, and urea levels alongside normal liver histology [93]. A phase I clinical trial reported no serious adverse effects following OPP supplementation at 450 mg GAE/day for 60 days in healthy volunteers [94]. While these findings are encouraging, the phase I trial involved only five participants, severely limiting its statistical power and generalisability. Larger, dose-escalation safety studies with broader participant cohorts are necessary before clinical translation can be meaningfully considered.

The current evidence base, while promising, is insufficient to draw firm mechanistic conclusions regarding the anti-angiogenic efficacy of oil palm-derived compounds in CRC. Key priorities for advancing this field include: (i) updated mechanistic studies using contemporary molecular tools, including transcriptomics, proteomics, and functional angiogenesis assays in physiologically relevant hypoxic models; (ii) isoform- and fraction-specific characterisation of lesser-studied constituents, including carotenoids, fatty acids, and flavonoids; and (iii) critically, spatially resolved analysis of compound distribution within CRC tumour tissues. The last of these priorities directly motivates the MSI-based framework proposed in this review. Determining whether POME-derived bioactive compounds localise to angiogenic niches and whether their spatial distribution correlates with the modulation of VEGF, HIF-1α, and NF-κB signalling would provide the mechanistic specificity that bulk analyses have thus far been unable to deliver and would constitute a substantive advance in the translational evaluation of nutraceutical anti-angiogenic candidates.

## 6. Mass Spectrometry as a Spatial Mapping Tool

Establishing whether POME-derived bioactive compounds modulate angiogenic signalling in CRC requires analytical platforms capable of resolving both molecular identity and spatial localisation within intact tumour tissues. Conventional approaches, including immunohistochemistry (IHC), immunofluorescence (IF), ELISA, and PCR-based gene expression analysis, provide valuable information on protein or transcript abundance but are fundamentally limited in spatial resolution and multiplexing capacity. Multiplexed IHC/IF typically requires serial tissue sections, exhausting limited biopsy material and complicating cross-marker comparisons [95], while bulk extraction methods such as PCR sacrifice spatial context entirely [96]. Critically, none of these approaches can determine whether an exogenous bioactive compound is present within a specific microenvironmental niche, such as a hypoxic region or area of active vascular remodelling, or whether its localisation correlates spatially with the angiogenic mediators it is proposed to modulate. This inability to resolve compound–pathway co-localisation represents the central analytical gap that motivates the framework proposed in this review.

**Figure 7:**
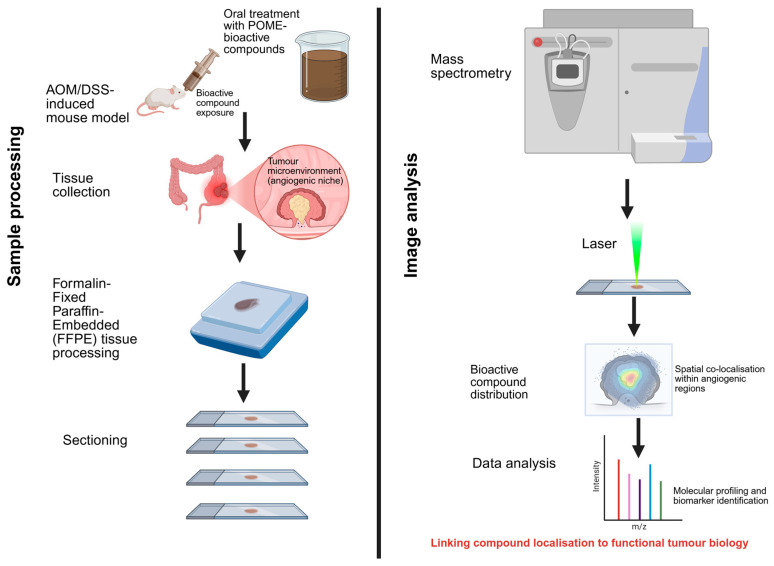
**Experimental workflow for spatially resolved analysis of POME−derived bioactive compounds using mass spectrometry imaging (MSI).** Schematic representation of a proposed experimental pipeline for investigating the spatial distribution and functional relevance of oil palm-derived bioactive compounds within colorectal tumour tissues. The workflow begins with an AOM/DSS−induced mouse model subjected to oral administration of POME−derived bioactive compounds, followed by tumour tissue collection, formalin−fixed paraffin-embedded (FFPE) processing, and tissue sectioning. Subsequent MSI analysis enables laser-based ionisation and detection of molecular species directly within tissue sections, generating spatial maps of bioactive compound distribution.

MSI addresses this gap directly by enabling label-free, spatially resolved detection of metabolites, lipids, peptides, and proteins within intact tissue sections [97,98]. MSI operates by scanning tissue in a raster pattern, acquiring a full mass spectrum at each pixel following surface ionisation. Molecules are separated by their mass-to-charge ratio (*m*/*z*), enabling simultaneous reconstruction of spatial distribution maps for multiple molecular species across a single tissue section. Molecular identification is achieved through accurate mass measurement, database matching, or structural confirmation via tandem MS/MS fragmentation (Figure 7) [99]. Critically, this capability allows simultaneous mapping of exogenous bioactive compounds alongside endogenous angiogenic markers, including VEGF, HIF-1α, and NF-κB pathway metabolites, within the same tissue section, enabling direct spatial correlation analysis that no other current platform provides.

Figure 7 illustrates an experimental workflow for investigating the spatial distribution of POME-derived bioactive compounds in colorectal tumour tissue using MSI, including preclinical modelling, tissue processing, and spatial molecular analysis. This experimental workflow provides the technical foundation for the conceptual framework proposed in this study, where MSI is positioned as a tool for linking spatial compound distribution to tumour-angiogenic function.

### 6.1. Established Applications Relevant to POME Research

The translational relevance of MSI to the present framework is supported by concrete precedents in cancer pharmacology and CRC research. MALDI-MSI has been used to characterise proteomic signatures across distinct regions of CRC tissues, revealing spatial heterogeneity in tumour architecture, extracellular matrix organisation, and cellular distribution [100], demonstrating that MSI can resolve region-specific molecular profiles directly relevant to angiogenic microenvironments. In pharmacological applications, MSI has been used to map the intratumoral distribution and penetration of chemotherapeutic agents, including paclitaxel, irinotecan, and erlotinib, within tumour tissues, identifying regions of limited drug exposure associated with therapeutic resistance [101]. These studies establish a direct methodological precedent for using MSI to track exogenous bioactive compounds, including POME-derived phenolics, tocotrienols, and carotenoids, within CRC tumour tissues following in vivo administration. Importantly, such drug-mapping studies have revealed that spatial heterogeneity of compound distribution, rather than bulk tissue concentration alone, determines functional outcome, a finding with direct implications for nutraceutical evaluation and providing the rationale for applying MSI to POME-derived compounds.

In the context of angiogenesis research specifically, MSI has been applied to map lipid species within tumour microenvironments, with DESI-MSI revealing distinct phospholipid profiles that distinguish endothelial, epithelial, and highly proliferative cell populations within tumour tissues [102,103]. These findings demonstrate that MSI can resolve molecular signatures within vascular and angiogenic niches, the exact spatial context in which POME-derived compound localisation must be evaluated to test the hypothesis advanced in this review.

### 6.2. Platform Selection and Complementarity

Three MSI ionisation platforms are relevant to the proposed research framework, each offering distinct and complementary analytical capabilities. MALDI-MSI enables detection of a broad range of biomolecules, including peptides, proteins, and lipids, with spatial resolution approaching 1 μm, making it well-suited for profiling endogenous angiogenic markers and larger POME-derived constituents such as tocotrienols and carotenoids within tumour tissue sections, though matrix application during sample preparation may introduce variability [104]. DESI-MSI operates under ambient conditions with minimal sample preparation, enabling rapid analysis of metabolites and small phenolic compounds, including gallic acid derivatives and hydroxycinnamic acids present in POME, with a spatial resolution of approximately 10 μm [105]. Its compatibility with FFPE tissue sections and ambient ionisation conditions makes it particularly practical for clinical and preclinical CRC specimens. SIMS-MSI offers the highest spatial resolution at the sub-micrometre scale, enabling the subcellular localisation of lipid species and elemental distributions, though its high-energy sputtering process restricts detection, primarily of small molecules and lipids [104]. For the proposed framework, a complementary multi-platform approach, using DESI-MSI for phenolic metabolite mapping, MALDI-MSI for protein and lipid co-localisation with angiogenic markers, and SIMS-MSI for subcellular lipid distribution, would provide the most comprehensive spatial characterisation of POME-derived compound behaviour within the CRC tumour microenvironment.

### 6.3. Technical Limitations and Mitigation Strategies

Several MSI limitations must be acknowledged. Sensitivity remains a key challenge, particularly for low-abundance nutraceutical compounds within compositionally complex tumour tissues, where ion suppression from abundant lipids and proteins may reduce the detection of target metabolites [106,107]. Quantitative MSI is technically demanding and not yet standardised across platforms, limiting the ability to report absolute compound concentrations within tissue regions. Additionally, structural isomers sharing identical *m*/*z* values may confound compound identification without complementary MS/MS confirmation. These limitations can be partially mitigated through optimised tissue preparation protocols, the use of stable isotope-labelled internal standards, and the integration of MSI data with the complementary liquid chromatography-mass spectrometry (LC-MS) profiling of matched tissue extracts. Acknowledging these constraints is essential for accurately interpreting spatially resolved data on POME-derived compounds and avoiding the overstatement of mechanistic conclusions. 

### 6.4. Integration with AI and Computational Analysis

The integration of MSI with artificial intelligence (AI) and advanced computational tools has substantially expanded its analytical scope. AI-assisted data processing enables pattern recognition across large, high-dimensional MSI datasets, enhancing signal detection, reducing noise, and enabling the unsupervised identification of molecularly distinct tissue regions [108,109,110]. In the context of the proposed framework, AI-driven spatial clustering could be used to identify angiogenic niches within CRC tumour sections based on endogenous molecular signatures, against which the distribution of POME-derived compounds could then be systematically correlated. Furthermore, machine learning models trained on MSI data have demonstrated the capacity for the predictive modelling of therapeutic response based on spatial biomarker patterns [111], suggesting that a comparable approach could be developed to predict the anti-angiogenic efficacy of POME-derived candidates based on their intratumoral distribution profiles. This positions MSI not merely as a descriptive mapping tool but as a hypothesis-generating and predictive platform aligned with the precision nutraceutical oncology framework proposed here.

Collectively, MSI provides the analytical foundation necessary to operationalise the central hypothesis of this review: the spatial localisation of POME-derived bioactive compounds within CRC tumour tissues is predictive of their functional anti-angiogenic activity. By enabling the direct co-localisation analysis of exogenous compounds with endogenous angiogenic markers within intact tissue architecture, MSI bridges the mechanistic gap between compound presence and biological function, which bulk analyses cannot address. The experimental workflow proposed, encompassing AOM/DSS preclinical modelling, oral compound administration, FFPE tissue processing, and multi-platform MSI analysis, provides a tractable and methodologically grounded pipeline for testing this hypothesis and advancing the translational evaluation of POME as a spatially informed anti-angiogenic bioresource.

## 7. Future Perspectives and Conceptual Framework

The preceding sections have established three converging lines of evidence: that CRC angiogenesis is a spatially regulated process governed by interconnected VEGF, HIF-1α, and NF-κB signalling networks; that POME contains chemically diverse bioactive constituents with reported but mechanistically undercharacterised anti-angiogenic properties; and that MSI provides the only current platform capable of simultaneously mapping exogenous compound distribution and endogenous angiogenic markers within intact tumour tissue architecture. The convergence of these lines motivates a central, testable hypothesis: that the spatial localisation of POME-derived bioactive compounds within CRC tumour tissues is predictive of their functional anti-angiogenic activity. This section articulates what testing this hypothesis would require, what it would reveal, and why it constitutes a meaningful advance for both nutraceutical oncology and sustainable bioresource utilisation.

### 7.1. Importance of Spatial Localisation

The current evidence on oil palm-derived bioactives establishes that these compounds can modulate angiogenic mediators under controlled in vitro conditions but provides no information about whether they reach, accumulate within, or functionally engage angiogenic niches in vivo. This is not a trivial gap. The CRC tumour microenvironment is characterised by steep oxygen gradients, spatially restricted immune infiltration, and heterogeneous vascular density, features that create distinct microenvironmental niches in which angiogenic signalling intensity varies considerably across distances of tens to hundreds of micrometres. A compound that suppresses VEGF expression in a homogeneous cell monolayer may fail to reach hypoxic cores where HIF-1α-driven VEGF transcription is most active or may accumulate preferentially in well-perfused stromal regions where its anti-angiogenic impact is limited. Conversely, a compound with modest bulk activity may exhibit potent localised effects within specific angiogenic hotspots that are obscured in bulk analyses. Without spatially resolved data, neither scenario can be distinguished, and the therapeutic prioritisation of candidate compounds remains speculative. Notably, the relationship between immune regulation, hypoxia-driven signalling, and angiogenesis during early colorectal tumour development, including the premalignant polyp stage, remains particularly undercharacterised, and spatially resolved investigation of this continuum represents an important frontier for the field.

### 7.2. Testable Hypotheses

The framework proposed here generates three discrete, falsifiable hypotheses, addressable using the experimental pipeline:

**Hypothesis** **1.** *POME-derived bioactive compounds, particularly phenolics and tocotrienols, will exhibit non-uniform intratumoral distribution following oral administration in AOM/DSS-induced CRC models, with preferential accumulation in regions of elevated vascular density and VEGF expression rather than uniform tissue distribution.*

**Hypothesis** **2.** *Spatial co-localisation of POME-derived compounds with HIF-1α and NF-κB signalling activity will be stronger in hypoxic tumour niches than in normoxic regions, and this co-localisation pattern will correlate with the localised suppression of downstream angiogenic mediators, including MMP-9 and IL-8.*

**Hypothesis** **3.** *The AI-assisted spatial pattern analysis of MSI datasets will identify compound-associated molecular signatures that predict anti-angiogenic response with greater accuracy than bulk tissue concentration measurements, establishing spatial distribution as an independent predictor of nutraceutical efficacy.*

Each hypothesis is independently testable, generates falsifiable predictions, and collectively constitutes a progressive mechanistic argument—from compound distribution to pathway co-localisation, to functional outcome prediction—that advances the field beyond current descriptive bioactivity profiling.

### 7.3. Proposed Research Roadmap

**Figure 8:**
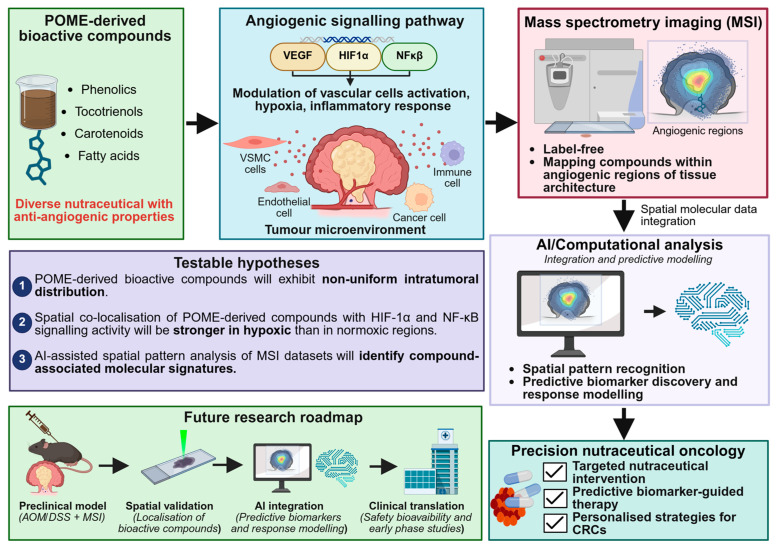
**Conceptual framework and future research roadmap for spatially resolved nutraceutical modulation of tumour angiogenesis using POME−derived bioactive compounds.** Schematic illustration of an integrative framework linking oil palm-derived bioactive compounds, angiogenic signalling pathways, and spatial molecular profiling using mass spectrometry imaging (MSI). POME-derived compounds, including phenolics, tocotrienols, carotenoids, and fatty acids, are proposed to modulate key angiogenic mediators such as vascular endothelial growth factor (VEGF), hypoxia-inducible factor−1α (HIF−1α), and nuclear factor−κB (NF−κB) within the tumour microenvironment. MSI enables label-free, spatially resolved mapping of bioactive compounds and their co-localisation with angiogenic signalling pathways in specific tumour regions, providing mechanistic insight into compound–pathway interactions. The framework further integrates artificial intelligence (AI) and computational analysis to facilitate spatial pattern recognition, biomarker discovery, and predictive modelling of nutraceutical response. Based on this integrative approach, several testable hypotheses are proposed, including the spatial enrichment of bioactive compounds in angiogenic hotspots and their co-localisation with key signalling mediators. A stepwise future research roadmap is outlined, beginning with preclinical validation using AOM/DSS-induced colorectal cancer models coupled with MSI analysis, followed by spatial validation of compound–pathway interactions, AI-driven biomarker identification, and eventual clinical translation. Collectively, this framework highlights a strategy for advancing precision nutraceutical oncology through spatially resolved and mechanistically informed investigation of tumour angiogenesis.

Translating these hypotheses into evidence requires a four-stage sequential pipeline (Figure 8). In Stage 1, the systematic chemical profiling of POME-derived fractions should be conducted using LC-MS and NMR to establish compound identity, concentration ranges, and stability under physiological conditions, with particular attention to the isoform-specific characterisation of fatty acids and quantification of phenolic subclasses most tractable for MSI detection. This addresses the critical gap regarding insufficient concentration and stability data and provides the chemical foundation for subsequent spatial studies. In Stage 2, the oral administration of characterised POME fractions in AOM/DSS-induced CRC mouse models should be followed by FFPE tissue collection and multi-platform MSI analysis, using DESI-MSI for phenolic metabolite mapping, MALDI-MSI for co-localisation with angiogenic protein markers, and SIMS-MSI for subcellular lipid distribution, to generate the first spatially resolved dataset linking POME-derived compound distribution to CRC tumour microenvironment architecture. In Stage 3, spatial co-localisation analysis between compound distribution maps and immunofluorescence-validated angiogenic markers should be performed, with AI-driven clustering used to identify microenvironmental niches where compound presence correlates with suppressed angiogenic signalling. Integration with transcriptomic and metabolomic datasets at this stage would further enable systems-level understanding of nutraceutical–tumour interactions and support the discovery of spatially resolved predictive biomarkers. This stage directly tests *Hypotheses 1 and 2* and provides the mechanistic evidence base currently absent from the literature. In Stage 4, compounds demonstrating spatially validated anti-angiogenic activity should advance to bioavailability optimisation, including nanoencapsulation or structural modification strategies to enhance tumour penetration, followed by early-phase clinical investigation assessing safety, pharmacokinetics, and target engagement in CRC patients. At this stage, the potential for POME-derived bioactives to complement conventional therapeutics through the synergistic modulation of interconnected angiogenic and inflammatory networks warrants particular investigation, as such combinations may broaden therapeutic impact while reducing treatment-related adverse effects [112].

### 7.4. Broader Implications for Precision Nutraceutical Oncology

If the central hypothesis is supported, the implications extend well beyond POME. The demonstration that spatial compound localisation predicts anti-angiogenic efficacy would establish a new evaluative paradigm for nutraceutical oncology (Figure 8), one in which spatial pharmacology, rather than bulk bioactivity, becomes the primary criterion for therapeutic prioritisation. This paradigm shift would fundamentally alter how nutraceutical candidates are screened, how preclinical models are designed, and how translational evidence is assembled for clinical development. It would also provide a rational basis for the spatial optimisation of compound delivery, directing bioactives toward specific tumour microenvironmental niches where their mechanistic impact is greatest, directly supporting the implementation of precision nutraceutical oncology, where dietary bioactive compounds are applied in a targeted and patient-specific manner based on tumour spatial and molecular profiles.

From a sustainability perspective, validating POME as a spatially active anti-angiogenic bioresource would establish a scientifically rigorous foundation for its valorisation within a circular bioeconomy framework, transforming the environmental liability of palm oil processing waste into a high-value, evidence-based medicinal resource. This dual contribution, to cancer biology and to sustainable industrial biotechnology, distinguishes the present framework from prior reviews and defines its unique scientific and translational value.

### 7.5. Outstanding Challenges

Realising this framework will require addressing several non-trivial challenges. The detection sensitivity of MSI for low-abundance nutraceutical compounds in complex tumour matrices remains a technical barrier, necessitating continued methodological development and the validation of quantitative MSI protocols. The translation from murine AOM/DSS models to human CRC, with its greater genetic and microenvironmental heterogeneity, will require careful model selection and parallel validation in patient-derived organoids or tumour explant systems. Standardised extraction and formulation protocols for POME-derived fractions are needed to ensure reproducibility across studies and to support dose-escalation safety assessments in clinical settings. Furthermore, the regulatory pathway for POME-derived nutraceutical candidates remains undefined; early engagement with regulatory frameworks governing botanical bioactives and functional food ingredients will be essential to facilitate clinical translation [113]. Well-designed clinical trials with clearly defined endpoints, appropriate patient stratification, and rigorous bioavailability assessment are ultimately required before any POME-derived compound can be meaningfully evaluated as a therapeutic or chemopreventive agent in CRC. These challenges are substantial but tractable, and their systematic resolution constitutes a clear and actionable forward agenda for the field.

Collectively, this framework reframes the evaluation of POME-derived bioactive compounds from descriptive bioactivity cataloguing toward spatially informed, mechanistically grounded, and clinically translatable investigation. By anchoring this agenda to a falsifiable central hypothesis and a sequentially testable research roadmap, the present review advances a strategic vision for precision nutraceutical oncology in CRC (Figure 8), one that is simultaneously responsive to the environmental imperative of sustainable palm oil waste management and to the scientific imperative of mechanism-based therapeutic development.

## 8. Conclusions

Oil palm-derived bioactive compounds, including tocotrienols, phenolic compounds, carotenoids, and fatty acids present in POME, represent a promising yet critically undercharacterised resource for anti-angiogenic intervention in colorectal cancer prevention and therapy. Accumulating evidence suggests that these compounds may modulate key molecular regulators of tumour angiogenesis, including VEGF, HIF-1α, and NF-κB, and influence broader signalling networks governing tumour microenvironment regulation. Reframing POME from an environmental burden into a reservoir of recoverable bioactive molecules therefore offers a compelling opportunity to integrate sustainable resource utilisation with biomedical innovation. Importantly, this reframing is not merely conceptual—the measurable concentrations of recoverable bioactives in raw POME, combined with demonstrated extraction feasibility, establish a practical foundation upon which a translational research agenda can be built.

Despite these promising observations, the current evidence base is constrained by its reliance in in vitro models, indirect mechanistic inference, and inconsistent findings across studies. The predominance of single-laboratory investigations limit independent replication, and the use of supraphysiological compound concentrations in many reported studies further temper the strength of existing conclusions. Critically, no study to date has examined how these compounds distribute within the spatially heterogeneous architecture of CRC tumour tissues or whether their localisation correlates with sites of active angiogenic signalling. This spatial dimension represents the central unresolved gap in the field, and its resolution is a prerequisite for establishing whether POME-derived compounds possess genuine therapeutic relevance in CRC.

This review proposes a spatially resolved nutraceutical oncology framework centred on the hypothesis that the spatial localisation of POME-derived bioactive compounds within CRC tumour tissues is predictive of their functional anti-angiogenic activity. Within this framework, MSI, across MALDI, DESI, and SIMS platforms, serves as a critical analytical bridge, linking compound distribution directly to angiogenic signalling dynamics within intact tumour microenvironments in a manner inaccessible to conventional bulk analyses. The four-stage research roadmap proposed here, from POME bioactive profiling through spatially resolved MSI mapping to clinically translatable validation, provides a sequentially testable pathway for converting this hypothesis into actionable evidence. This framework is distinguished from prior oil palm and POME reviews not only by its spatial analytical focus but by its insistence on falsifiable hypothesis generation and mechanistically grounded evaluation, standards that the field must adopt if nutraceutical oncology is to achieve genuine translational credibility.

Looking forward, the integration of MSI with AI, advanced computational tools, and multi-omics approaches will further enhance our ability to resolve spatial angiogenic signatures, identify predictive biomarkers, and stratify therapeutic responses to nutraceutical intervention. Such advances support the emergence of precision nutraceutical oncology, where bioactive compounds are applied strategically based on tumour spatial and molecular profiles rather than bulk bioactivity alone. The dual contribution of this agenda, advancing mechanistic cancer biology while providing scientific justification for sustainable palm oil waste valorisation, positions POME-derived nutraceutical research at the genuinely productive intersection of environmental responsibility and therapeutic innovation. Ultimately, multidisciplinary research combining sustainable bioresource valorisation, molecular oncology, and spatial analytical technologies will be essential for translating POME-derived bioactive compounds into effective, evidence-based strategies for colorectal cancer prevention and therapeutic intervention. The framework proposed here is intended not as a definitive answer but as a rigorous starting point; one that sets the methodological and conceptual standards necessary to determine whether POME-derived bioactives can fulfil their therapeutic promise in colorectal cancer.

## 9. Literature Search Strategy

This review is based on a narrative synthesis of the current literature related to oil palm-derived bioactive compounds, angiogenesis in colorectal cancer, and mass spectrometry imaging (MSI). Relevant studies were identified through searches in databases, including Google Scholar and CrossRef, using combinations of keywords such as “POME”, “oil palm bioactive compounds”, “angiogenesis”, “colorectal cancer”, and “mass spectrometry imaging”. Priority was given to studies that provide mechanistic insight, spatial biological relevance, or translational significance in the context of tumour microenvironment research. The selected literature was synthesised to support the conceptual framework and future perspectives proposed in this review.

## Figures and Tables

**Table 1:** Summary of major bioactive compounds present in palm oil mill effluent (POME) and their reported biological activities, with potential nutraceutical, biomedical, and industrial applications. These components include tannins, lignin, phenolic compounds, carotenoids, pectin, vitamin E, and fatty acids. Previous studies have demonstrated that these compounds possess diverse biological functions, including antioxidant, anti-inflammatory, anti-cancer, anti-angiogenic, and cardioprotective effects. In addition, several POME-derived constituents show potential translational value in pharmaceutical development, cosmeceutical formulations, nanomaterials, and functional food products.

POME-Derived Bioactive Components	Biological Mechanism and Potential Translational Value
Tannins [31]	Anti-parasite drugs (Anthelmintics) [32] Antioxidant, anti-inflammatory, and cardioprotective properties [33]
Lignin [34]	Nanomaterial products such as drug-loaded microcapsules [35] Sunscreen/SPF booster [36]
Phenolics [37]	Prevent type 2 diabetes mellitus [38] Anti-cancer drug [39] Anti-angiogenesis properties [40]
Carotene [41]	Anti-oxidant and anti-aging properties [42] Anti-inflammation properties [43] Cosmeceutical product [44]
Pectin [45]	Pectin hydrogel in food industry [46] Anti-inflammatory benefit [47]
Vitamin E [29]	Anti-atherogenic effects [48] Anti-inflammatory [49] Greater endothelial benefits of endothelial cells (ECs) [50] Maintains vascular integrity
Fatty acids [51]	Anti-aging related to vascular dysfunction [52] Modulate angiogenesis [53] Drugs with pro/anti-angiogenesis properties depending on isoform of fatty acids

## Data Availability

No new data were created or analysed in this study.
